# Small (≤ 20 mm) ground-glass opacity pulmonary lesions: which factors influence the diagnostic accuracy of CT-guided percutaneous core needle biopsy?

**DOI:** 10.1186/s12890-022-02058-z

**Published:** 2022-07-07

**Authors:** Yang Li, Chao Feng Yang, Jun Peng, Bing Li, Chuan Zhang, Jin Hong Yu

**Affiliations:** 1 Sichuan Key Laboratory of Medical Imaging, Department of Radiology, The Affiliated Hospital of North Sichuan Medical College, 63 Wenhua Road, Nanchong City, 637000 Sichuan Province China; 2 Department of Radiology, The People’s Hospital of Yuechi County, 22 East Jianshe Road, Yuechi County, 638350 Sichuan Province China; 3 Sichuan Key Laboratory of Medical Imaging, Department of Ultrasound, The Affiliated Hospital of North Sichuan Medical College, 63 Wenhua Road, Nanchong City, 637000 Sichuan Province China; 4 Department of Ultrasound, The People’s Hospital of Yuechi County, 22 East Jianshe Road, Yuechi County, 638350 Sichuan Province China

**Keywords:** Computed tomography, Diagnostic accuracy, Ground-glass opacity, Interventional radiology, Lung

## Abstract

**Background:**

The diagnostic accuracy of computed tomography (CT)-guided percutaneous core needle biopsy (CNB) for small (≤ 20 mm) ground-glass opacity (GGO) lesions has not been reported in detail.

**Objectives:**

To evaluate factors that affect the diagnostic accuracy of CT-guided percutaneous CNB for small (≤ 20 mm) GGO pulmonary lesions.

**Methods:**

From January 2014 to February 2018, 156 patients with a small (≤ 20 mm) GGO pulmonary lesion who underwent CT-guided CNB were enrolled in this study. Factors affecting diagnostic accuracy were evaluated by analyzing patient and lesion characteristics and technical factors. Significant factors were identified by multivariate logistic regression.

**Results:**

The diagnostic accuracy of CT-guided percutaneous CNB was 90.4% for small (≤ 20 mm) GGO pulmonary lesions. The diagnostic accuracy was higher for larger lesions (72.5% for lesions ≤ 10 mm, 96.6% for lesions between 11 and 20 mm [*P* < 0.001]). The diagnostic accuracy of CT-guided percutaneous CNB was 74.5% for lesions with > 90% GGO components and 97.2% for lesions with 50–90% GGO components (*P* < 0.001). In multivariate analysis, the significant factors influencing diagnostic accuracy were lesion size (*P* = 0.022; odds ratio [OR] for a lesion between 11 and 20 mm in size was approximately 5 times higher than that for a lesion ≤ 10 mm; 95% confidence interval [CI], 1.3 to 18.5), and GGO component (*P* = 0.015; OR for a lesion with 50–90% GGO components was approximately 6 times higher than that for a lesion with > 90% GGO components; 95% CI: 1.4 to 25.7).

**Conclusions:**

Lesion size and GGO component are factors affecting diagnostic accuracy. The diagnostic accuracy was higher for larger lesions and lesions with 50–90% GGO components.

## Introduction

Ground-glass opacity (GGO) is a nonspecific finding that can be caused by a variety of pulmonary lesions, including focal fibrosis, inflammatory diseases, atypical adenomatous hyperplasia (AAH), adenocarcinoma in situ (AIS), adenocarcinoma and bronchoalveolar carcinoma (BAC) [1, 2]. With the wide application of high-resolution CT (HRCT) for pulmonary lesions, the detection rate of GGO lesions has gradually increased, which has brought a new problem for the management of GGO lesions. The morphological findings of AAH and AIS show no significant difference from invasive adenocarcinoma on diagnostic HRCT [3]. Therefore, obtaining a definitive diagnosis is considered to be an important step in the management and treatment of GGO lesions.

CT-guided percutaneous core needle biopsy (CNB) is an indispensable method in the histologic diagnosis of GGO lesions, and achieves high diagnostic accuracy with acceptable complication rates [2, 4–6]. The component percentage and sizes of GGO lesions vary, resulting in technical and diagnostic difficulties. Unfortunately, because previous studies have included a relatively limited number of patients, the diagnostic accuracy results reported by these studies were variable and often contradictory [2, 4, 5, 7]. For example, in a study by Kim [2], the diagnostic accuracy of biopsies for GGO-dominant lesions showed no significant difference between lesions with different sizes and GGO components. In a report of 47 core biopsies for GGO-dominant lesions, as the size of the lesion increased, the diagnostic accuracy tended to increase [5]. Yamagami et al. reported that the diagnostic accuracy was influenced by the GGO component [7]. An extensive analysis of which parameters affect the diagnostic accuracy of CT-guided CNB for small (≤ 20 mm) GGO lesions may be useful for performing this technique during daily practice. Thus, in this retrospective study, we assessed lesion characteristics and technical factors that affected the diagnostic accuracy of CT-guided CNB for small (≤ 20 mm) GGO lesions.

## Methods

### Patient selection

**Fig. 1:**
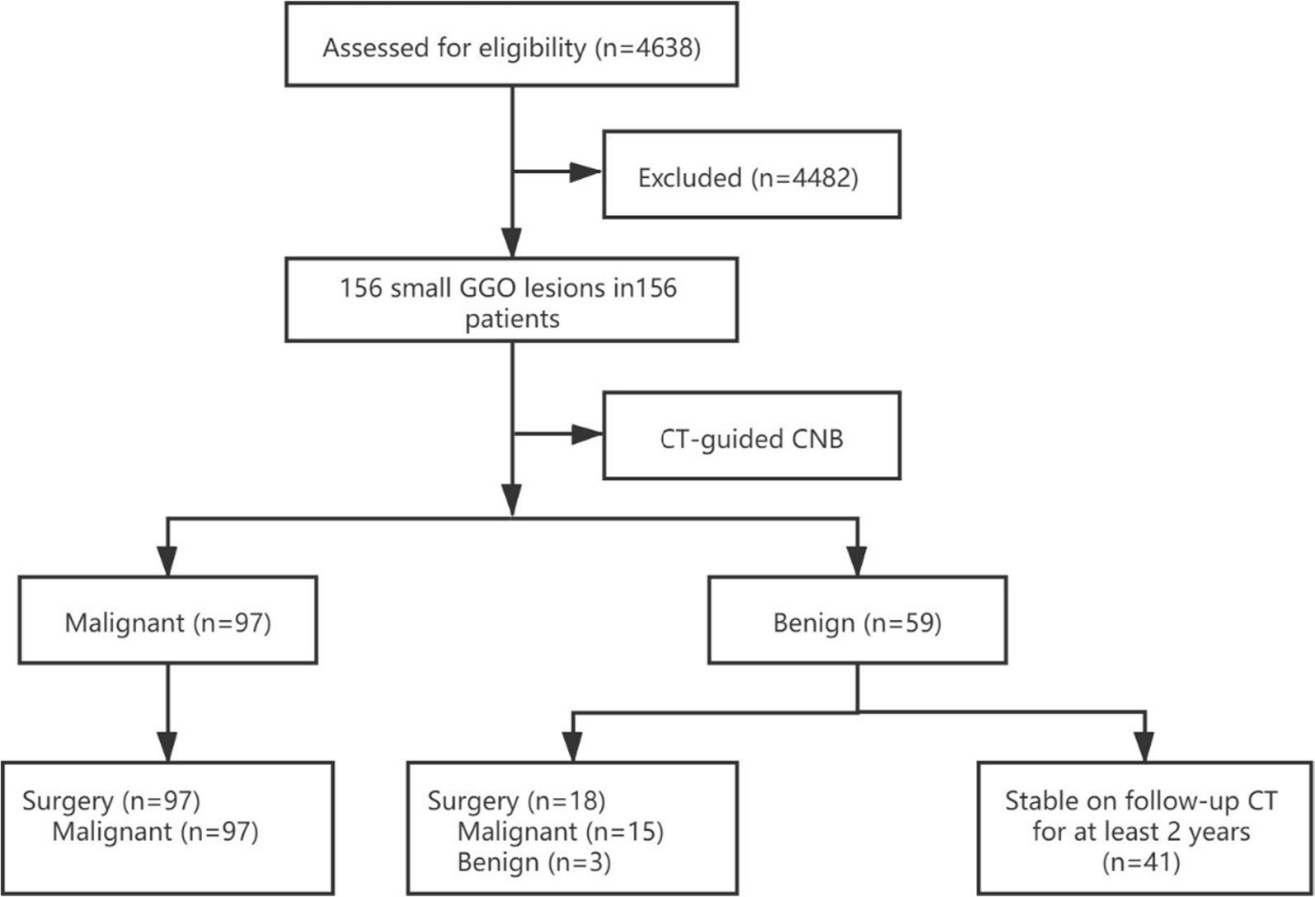
Flow chart of the study group

A total of 4638 CT-guided CNB procedures for pulmonary lesions were performed at our institution between January 2014 and February 2018 (Fig. 1). Two experienced chest radiologists identified GGO lesions on HRCT by consensus. GGO was defined as a focal area in the lung with increased attenuation and preservation of the vessels and bronchi margins [2, 6]. The percentage of the GGO component was calculated as follows: ([D_GGO_ – D]/D_GGO_) × 100, where D_GGO_ is the greatest diameter of the lesion (including the GGO area), and D is the greatest diameter of the lesion without GGOs [2]. The exclusion criteria were lesions with a less than 50% GGO component (because these lesions are mainly solid in nature) and lesions less than 5 mm in maximum diameter. Finally, 156 small (≤ 20 mm) GGO lesions in 156 patients (65 men, 91 women; range: 22–82 years, mean age of 54.0 ± 12.5 years) with a final diagnosis were included in this study. The mean diameter of the GGO lesions was 14.2 ± 4.4 mm (range, 5–20 mm). This retrospective study was approved by the institutional review board of the Affiliated Hospital of North Sichuan Medical College (Approval Number: K2021168). The requirement for informed consent was waived owing to the retrospective study design.

Coagulation factors were checked before the CNB procedure. None of the patients took any anticoagulants or platelet inhibitors for at least 1 week.

### Biopsy procedure

The CNB procedures were performed under guidance with a multislice helical CT scanner (MX 16-slice, Philips and Neusoft Medical Systems, China) and by three experienced chest radiologists with 5, 6 and 8 years of experience in chest imaging and intervention. The patients were placed in supine, prone, or lateral decubitus positions, depending on the location of the lesion as well as the patients' body habitus. CT images were obtained in the area of interest with 3 mm thin-section contiguous images that were to be used to plan the needle path to avoid bullae, emphysema, vascular structures, and fissures in the planned pathway as much as possible. Approximately 3 ml of 1% lidocaine was injected into the subcutaneous tissue at the puncture site.

Biopsy procedures were performed using the standard coaxial technique. Breath holding was limited to when a 19-gauge coaxial needle (TruGuide, Bard, Tempe, AZ, USA) crossed the pleura, and a single pleural puncture was performed in all cases. When the coaxial needle was inserted into the lesion, the stylet was removed, and a core biopsy specimen was obtained with a matching 20-gauge cutting needle (Magnum Needles, Bard, Tempe, AZ, USA) and biopsy gun (Magnum, Bard, Tempe, AZ, USA). The throw length was selected according to lesion size and anatomical location. Decisions to obtain additional specimens depended on whether the specimen was sufficient for histological evaluation by visual inspection. The standard practice was to obtain two to three specimens at our institution. Subsequent specimens were obtained from different areas within the lesion by manually moving the outer needle for random sampling. Each specimen was immersed in a 10% formalin-filled container. After the biopsy, 1–3 mL of normal saline was instilled into the needle track during extraction of the coaxial sheath [8].

CT images were obtained immediately after the procedure to evaluate for the presence of procedural complications, such as pneumothorax and hemorrhage. Patients were admitted into the wards for observation, and underwent a follow-up chest radiograph six hours later, or sooner if the patient became symptomatic. A chest tube was placed for drainage in patients with pneumothorax that was enlarging or accompanied by symptoms of shortness of breath, dyspnea, or low oxygen saturation.

### Data collection

Factors that may affect the outcome were recorded. Age, sex and emphysema detected on CT were considered to be patient characteristics. The degree of background emphysema was divided semiquantitatively into none, mild, moderate, or prominent [9]. The lesion characteristics included lesion size, percentage of the GGO component, lesion depth and lesion location. Lesion size was defined as the greatest diameter of the lesion in the thin-section CT lung window settings. The lesions were divided into two groups on the basis of size: ≤ 10 mm and 11–20 mm. The lesions were divided into two categories according to the percentage of the GGO component (> 90% vs. 50–90%). Lesion depth was defined as the length of the aerated lung traversed by the biopsy needle. The technical factors included specimen size, number of specimens and individual radiologist. The length of each specimen was documented by the radiologist. The specimens were divided into two categories based on the length of each specimen: < 15 mm and ≥ 15 mm.

### Analysis of CNB results

A positive CNB result was considered as a true-positive if surgical confirmation was obtained. A negative CNB result was considered as a true-negative if surgical confirmation or a definitive benign diagnosis was obtained and if the lesion subsequently resolved or showed no change in size on follow-up CT for at least 2 years. A positive finding was considered as a false-positive if the surgical resection yielded a benign result, or if lesion resolution was observed on follow-up CT in the absence of therapy. A negative finding was considered as a false-negative if a malignant diagnosis was confirmed by surgical resection and if the lesion increased in size.

### Statistical analysis

The chi-square test or Fisher’s exact test was used to analyze categorical variables, and the Mann–Whitney U test was used for continuous variables. Candidate variables with a *p* value less than 0.2 in the univariate analyses were entered in multivariate logistic regression. SPSS software (SPSS, Chicago, IL, USA) was used for statistical analysis. A *p* value less than 0.05 was considered significant.

## Results

**Fig. 2:**
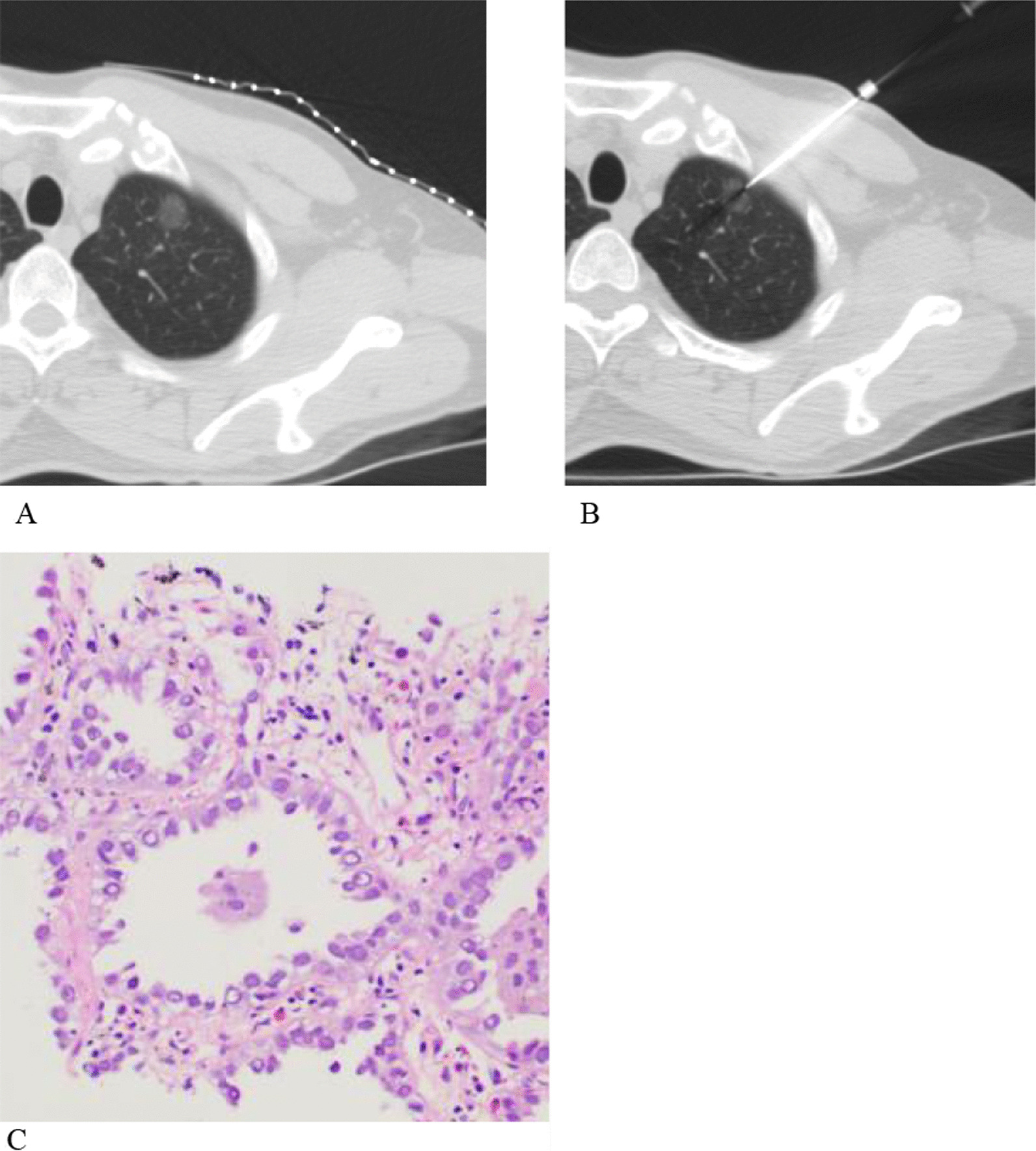
CT-guided core biopsy in 47-year-old man with pure ground-glass opacity (GGO) lesion in left upper lobe. **A**. CT scan shows 10-mm pure GGO lesion in left upper lobe. **B**. CT scan obtained during biopsy shows needle targeting GGO lesion. **C**. Histologic diagnosis of biopsy was adenocarcinoma. (H and E, × 200)

Adequate biopsy specimens were obtained for histological analysis in all lesions (Fig. 2). There were 112 malignant lung lesions and 44 benign lung lesions; the final diagnoses of the GGO lesions are summarized in Table 1. Ninety-seven malignant lesions identified by CNB were surgically resected for confirmation, including adenocarcinomas (n = 90) and bronchoalveolar carcinomas (n = 7). Of the 18 lesions negative for malignancy on CNB, 11 lesions did not decrease in size, whereas seven lesions increased in size, and these lesions were confirmed to have a definitive diagnosis by surgical resection. Of these lesions, 15 were confirmed as adenocarcinomas, and the other three lesions were revealed to be benign, including interstitial fibrosis (n = 2) and organizing pneumonia (n = 1). The sensitivity, specificity, accuracy, and positive and negative predictive values of CT-guided CNB in the diagnosis of 156 small (≤ 20 mm) GGO lesions were 86.6%, 100%, 90.4%, 100% and 74.6%, respectively (Table 2).

**Table 1:** Histologic results for 112 malignant lung lesions and 44 benign lung lesions with final diagnostic

Diagnosis	Number of cases
Adenocarcinoma	105
Bronchioalveolar carcinoma	7
Non-specific inflammation	24
Interstitial fibrosis	11
Organizing pneumonia	7
Tuberculosis	2
Total	156

**Table 2:** Final diagnoses

CNB histologic results	Final diagnosis		Total
	Malignant	Benign	
Malignant	97	0	97
Benign	15	44	59
Total	112	44	156

Diagnostic accuracy in relation to the various factors of the 156 biopsy lesions are detailed in Table 3. From initial univariate analyses, there was a significant difference in diagnostic accuracy between lesions with different sizes (*P* < 0.001) and GGO component percentages (*P* < 0.001). The diagnostic accuracy was higher for larger lesions; it was 72.5% (29 of 40) for lesions 10 mm or smaller and 96.6% (112 of 116) for lesions between 11 and 20 mm.

**Table 3:** Diagnostic accuracy in relation to various factors

Variable	No. of diagnostic cases	Total No. of cases	Diagnostic accuracy (%)	*P* value
Age (year)				0.652
< 50	65	71	91.5	
≥ 50	76	85	89.4	
Sex				0.215
Male	61	65	93.8	
Female	80	91	87.9	
Emphysema on CT				0.972
None	101	112	90.2	
Mild	19	21	90.5	
Moderate	14	15	93.3	
Prominent	7	8	87.5	
Lesion size (mm)				< 0.001
≤ 10	29	40	72.5	
11–20	112	116	96.6	
GGO component				< 0.001
> 90%	35	47	74.5	
50–90%	106	109	97.2	
Lesion depth (mm)				
0	35	38	92.1	0.891
1–20	48	53	90.6	
> 20	58	65	89.2	
Lesion location				0.331
Upper and middle lobes	93	101	92.1	
Lower lobe	48	55	87.3	
Specimen size (mm)				0.491
< 15	81	91	89.0	
≥ 15 mm	60	65	92.3	
Individual radiologist				0.788
A	39	42	92.9	
B	55	61	90.2	
C	47	53	88.7	

A significantly higher diagnostic accuracy (97.2% [106/109]) was observed for lesions with 50–90% GGO components than for lesions with > 90% GGO components (74.5% [35/47]). However, other factors, including age, sex, emphysema on CT, lesion depth, lesion location, specimen size and individual radiologist, had no statistically significant effect on diagnostic accuracy (Table 3). The diagnostic accuracies were further analyzed in subgroups according to lesion size and GGO component, as shown in Table 4. The difference in diagnostic accuracy among the different percentages of GGO components was more marked for lesions 10 mm or smaller than for lesions between 11 and 20 mm. Among lesions with > 90% GGO components, the diagnostic accuracy for lesions 11–20 mm in size was considerably higher than that for lesions ≤ 10 mm (*p* = 0.014). The diagnostic accuracy for lesions with a 50–90% GGO component increased with lesion size; however, the improvement in accuracy was not statistically significant (*p* = 0.078).

**Table 4:** The diagnostic accuracy based on lesion size and GGO component

Variable	Diagnostic accuracy*		*p*-value
	> 90% GGO component (n = 47)	50–90% GGO component (n = 109)	
Lesion size (mm)			
≤ 10 (n = 40)	12/21 (57.1%)	17/19 (89.5%)	0.022
11–20 (n = 116)	23/26 (88.5%)	89/90 (98.9%)	0.035
*P* value	0.014	0.078	

*Data are numbers of patients, with percentages in parentheses

Because some factors may explain other factors, resulting in indirect bias, we used multivariate logistic regression to estimate odds ratios (ORs) for diagnostic accuracy. The significant candidate variables were (a) lesion size and (b) GGO component. For lesion size, there was an approximately 5.0 times higher likelihood of obtaining a definitive diagnosis (*P* = 0.022; OR = 4.8; 95% confidence interval [CI]: 1.3–18.5) with an increase in lesion size subgroup (≤ 10 mm to 11–20 mm) (Table 4). For the GGO component, a definitive diagnosis was approximately 6 times more likely to be obtained for a lesion with 50–90% GGO components than for a lesion with > 90% GGO components (*P* = 0.015; OR = 6.0; 95% CI: 1.4–25.7) (Table 5).

**Table 5:** Diagnostic accuracy to various related factors evaluated by multiple logistic regression

Variable	Diagnostic accuracy (%)	*P* value	OR	95% CI
Lesion size (mm)				
≤ 10	72.5 (29/40)	Reference	1.0	
11–20	96.6 (112/116)	0.022	4.8	1.3–18.5
GGO component				
> 90%	74.5%(35/47)	Reference	1.0	
50–90%	97.2%(106/109)	0.015	6.0	1.4–25.7

Regarding biopsy-induced complications, pulmonary bleeding was the most common complication in the present study and CT images acquired immediately after the biopsy showed pulmonary bleeding in 75 (48.1%) of the 156 procedures. Furthermore, alveolar hemorrhage occurred in all patients with lesions with > 90% GGO components; among them, fifteen patients experienced hemoptysis after the biopsy. All bleeding complications were treated conservatively. Pneumothorax occurred in 11 (7.1%) of the 156 cases. Immediate manual aspiration was performed in three of these cases, and chest tube placement was required in one of these cases (0.6%). No patients developed air emboli with clinical symptoms.

## Discussion

With the prevalence of low-dose HRCT for screening, the detection rate of small lung cancer, especially peripheral small adenocarcinoma that frequently shows focal GGOs, has been increasing [10, 11]. Kim et al. reported that approximately 75% of persistent pulmonary GGO lesions are attributed to bronchioloalveolar cell carcinoma (BAC) or adenocarcinoma with a predominant BAC component [12]. Si et al. found that the presence of a GGO lesion > 7.5 mm increases the possibility of minimally invasive adenocarcinoma (MIA) [13]. Our results showed that CT-guided percutaneous CNB can be a relatively safe and accurate diagnostic technique for small (≤ 20 mm) GGO pulmonary lesions with acceptable complication rates. Of the 112 lesions diagnosed as malignancy, 105 were adenocarcinomas and 7 were bronchioalveolar carcinomas, as shown in a previous study [6]. In the present study, the sensitivity, specificity, accuracy, and positive and negative predictive values of CT-guided CNB for diagnosing 156 small (≤ 20 mm) GGO lesions were 86.6%, 100%, 90.4%, 100% and 74.6%, respectively. Furthermore, the diagnostic accuracy was significantly influenced by lesion size and GGO component.

To the best of our knowledge, few studies have reported on the various factors affecting the diagnostic accuracy of CT-guided biopsy for GGO lesions. Regarding lesion size, our present study found that the diagnostic accuracy tended to be lower for smaller lesions (≤ 10 mm). The result based on lesion size suggests that it is difficult to target smaller lesions, leading to a greater chance of sampling error. It is necessary to exercise caution in the interpretation of small GGOs negative for malignancy. Kim et al. [2] reported the diagnostic accuracy of biopsies for GGO-dominant lesions (GGO component ≥ 50%; n = 46) to be 91% for lesions < 2 cm and 96% for lesions ≥ 2 cm (*P* = 1.0). Hur et al. [4] described the outcomes of 28 aspiration biopsies for GGO-dominant lesions and reported a diagnostic accuracy of 80% for lesions ≤ 10 mm, 80% for those 11–20 mm, and 88% for those > 20 mm in size (*P* > 0.05). Yamauchi et al. [5] reported the outcomes of 47 core biopsies for GGO-dominant lesions and found that the diagnostic accuracy tended to increase with an increase in lesion size (88% for lesions ≤ 10 mm, 98% for those 11–20 mm, and 100% for those > 20 mm). Shimizu et al. [11] found a similar trend for the diagnostic accuracy of CT-guided fine needle aspiration for small (≤ 2 cm; n = 46) GGO-dominant lesions. The diagnostic accuracy of CT-guided fine needle aspiration was 35.2% for lesions ≤ 10 mm, 50.0% for lesions 11–15 mm, and 80.0% for lesions 16–20 mm, which suggests that it is difficult to obtain an accurate diagnosis for smaller lesions. In the present study, eleven cases negative for malignancy occurred in lesions ≤ 10 mm and nine in lesions 11–20 mm, and there was an approximately 5.0 times higher likelihood of obtaining a definitive diagnosis (OR = 4.8; 95% CI: 1.3–18.5) with an increase in lesion size subgroup. These different results can be explained by the use of different study populations and different types of needles. First, our study included 156 small GGO lesions, and the mean lesion diameter was 14.2 ± 4.4 mm (range, 5–20 mm). Second, compared with needle aspiration cytology, core biopsy may be more effective in the diagnosis of GGO lesions because it can obtain enough samples for pathological diagnosis and molecular analysis [5, 14]. Recently, an augmented reality navigation system has been introduced for percutaneous CT-guided pulmonary GGO biopsies. Faiella et al. reported that this system can increase the diagnostic success rate for GGO lesions < 1.5 cm [15].

When comparing diagnostic accuracy according to the GGO component, the difference was statistically significant between groups. We saw a decrease in diagnostic accuracy with an increase in the percentage of the GGO component. A confirmed diagnosis was obtained in only 74.5% of lesions with > 90% GGO components, as opposed to 97.2% of lesions with 50–90% GGO components. Our result was similar to previous reports [4, 7]. According to Hur et al., the diagnostic accuracy for the 7 lesions with > 90% GGO components was 57%, while that for the 21 lesions with 50–90% GGO components was 90% [4]. Yamagami et al. also reported that the diagnostic accuracy was significantly influenced by the percentage of GGO component. The diagnostic accuracy was found to be 73.9% for pure GGO lesions and 96.8% for part-solid GGO lesions [7]. However, Kim et al. [2] reported conflicting results; the diagnostic accuracy (91%) for lesions with > 90% GGO components was not significantly different from that (92%) for lesions with 50–90% GGO components. In that study, the mean size of the GGO lesions was larger than those in our study (19.0 ± 0.9 mm vs. 14.2 ± 4.4 mm). Kim et al. also found that lesions with > 90% GGO components were more likely to result in nondiagnostic samples than lesions with 50–90% GGO components and suggested that it is necessary to conduct further studies to evaluate the diagnostic accuracy of CNB according to percentage of GGO components. Our results showed that false-negative results were more frequent in lesions with > 90% GGO components (n = 12) than in lesions with 50–90% GGO components (n = 3). A definitive diagnosis was approximately 6 times more likely to be obtained for a lesion with 50–90% GGO components than for a lesion with > 90% GGO components (OR = 6.0; 95% CI: 1.4–25.7). Inoue et al. [6] reported that parenchymal hemorrhage always occurred in biopsy of pure GGO (> 95% GGO components) lesions. Our results are in agreement with their study, and this may be related to the preservation of bronchus and pulmonary vessels in GGO lesions. Bleeding caused by the cutting of pulmonary vessels in these lesions may spread through the airway to the surrounding parenchyma, and alveolar hemorrhage occurred in all patients with lesions with > 90% GGO components. Because parenchymal hemorrhage can obscure GGO lesions, false-negative results are more common for lesions with > 90% GGO components than for lesions with 50–90% GGO components.

The incidence of pneumothorax following lung biopsy with CT guidance has been reported as 6.2–69% and that of chest tube placement as 0.5–38% [9, 16–19]. In our study, the rate of pneumothorax was 7.1% and that of chest tube placement was 0.6%, which were similar to the reported rates in previous studies. Our study showed that biopsy of lesions with > 90% GGO components always resulted in parenchymal hemorrhage, which was similar to a previous study [6]. This may be related to the preservation of the vessels and bronchi in GGO lesions. The bleeding caused by cutting the vessels in GGO lesions may spread to the surrounding parenchyma through the airway.

Our study had several limitations. First, the retrospective design may have resulted in selection bias. Second, a relatively small number of patients with lesions with > 90% GGO components (47/156) were included. The diagnostic accuracy should be further validated with a larger number of cases. Last, although the follow-up duration was at least 2 years after the biopsy, pure small GGOs show slow growth and have been reported to have a volume-doubling time of greater than 800 days in 20–50% of cases [20–22].

In conclusion, the lesion-related characteristics affecting the diagnostic accuracy of CNB for small (≤ 20 mm) GGO lesions were identified. The results of our study suggest that the diagnostic accuracy was higher for larger lesions and lesions with 50–90% GGO components.

## Data Availability

The datasets during and/or analyzed during the current study available from the corresponding author on reasonable request.
